# Ineffective Antibiotic Prophylaxis: An Unusual Presentation of Infective Endocarditis with Insights into the Appropriateness of Prophylaxis

**DOI:** 10.7759/cureus.4860

**Published:** 2019-06-07

**Authors:** Hiren Patel, Sundeep Kumar, Nway L Ko Ko, Jelena Catania, Aamir Javaid

**Affiliations:** 1 Internal Medicine, University of Central Florida College of Medicine, Orlando, USA; 2 Internal Medicine - Infectious Diseases, University of Central Florida College of Medicine, Orlando, USA; 3 Cardiology, University of Central Florida College of Medicine/Hospital Corporation of America Graduate Medical Education Consortium, Orlando, USA

**Keywords:** aortic valve, endocarditis, antibiotic prophylaxis, streptococcus intermedius, streptococcus spp

## Abstract

The practice of antibiotic prophylaxis for the prevention of infective endocarditis (IE) has undergone a major paradigm shift over the past few decades. From the earlier practice of antibiotic prophylaxis for all patients undergoing dental procedures, since 2006, the American College of Cardiology/American Heart Association (ACC/AHA) guidelines have now narrowed down the indications to use it only in patients at high risk for IE. A true concern for antibiotic resistance remains eminent when used without appropriate indications. Through this case presentation of IE and the associated preceding use of antibiotics, we take this opportunity to explain the appropriateness of prophylaxis in patients from different risk groups. We also shed some light on alternative, effective, and, yet, harmless measures for the prevention of IE in patients at low risk.

## Introduction

Antibiotics for the prevention of infective endocarditis (IE) have been in routine clinical practice without much supportive clinical data until the publication of the 2006 American College of Cardiology/American Heart Association (ACC/AHA) guidelines limiting indications for their role [1]. Updated guidelines over 10 years later still stress that antibiotic prophylaxis for IE should only be used in high-risk individuals [2]. Prophylactic antibiotics can fail even when the infecting organism is susceptible to antibiotics and the number needed to treat for the prevention of the very few cases of IE is relatively high [3]. In spite of the guidelines, epidemiological studies reveal the inappropriate use of antibiotics for infective endocarditis prophylaxis, including but not limited to incorrect dosing, timing, and choice of antibiotics [4-5].

Transient bacteremia can commonly follow dental procedures but do not always necessarily require gingival manipulation. It can also potentially occur at a low grade/intensity with inadequate dental hygiene. Reduction in bacteremia with antibiotic prophylaxis has not prevented IE significantly [6]. In fact, inappropriate antibiotic use in such cases can lead to antibiotic resistance [2].

According to the ACC/AHA Task Force as of 2017, there have been no randomized controlled trials performed to show the benefit of antibiotic prophylaxis in low-risk patients [2]. A key concern is educating patients about the appropriate indications, dosing, and timing of effective antibiotic prophylaxis prior to the procedure. Adequate dental hygiene is important to prevent continuous low-grade bacteremia (potential etiology for IE) with effective, high-potency mouthwashes and optimum oral hygiene to control viridans group streptococci.

## Case presentation

A 53-year-old African American female with well-controlled non-insulin dependent diabetes mellitus (hemoglobin A1c 5.8) presented to the emergency department with a four-week history of generalized fatigue, malaise, and 20 lb weight loss, along with a two-week history of right upper quadrant pain, subjective fever, nausea, and vomiting. The patient underwent dental root extraction five weeks prior to admission. Although she did not have a prior history of endocarditis or a prosthetic valve, she was given one dose of two grams amoxicillin for endocarditis prophylaxis, which she took the day before the procedure. On arrival, the patient was in sepsis, with a temperature of 101.9° F and blood pressure of 96/60 mm Hg but attained hemodynamic stability after appropriate fluid resuscitation. Physical examination was significant for tender hepatomegaly.

**Figure 1:**
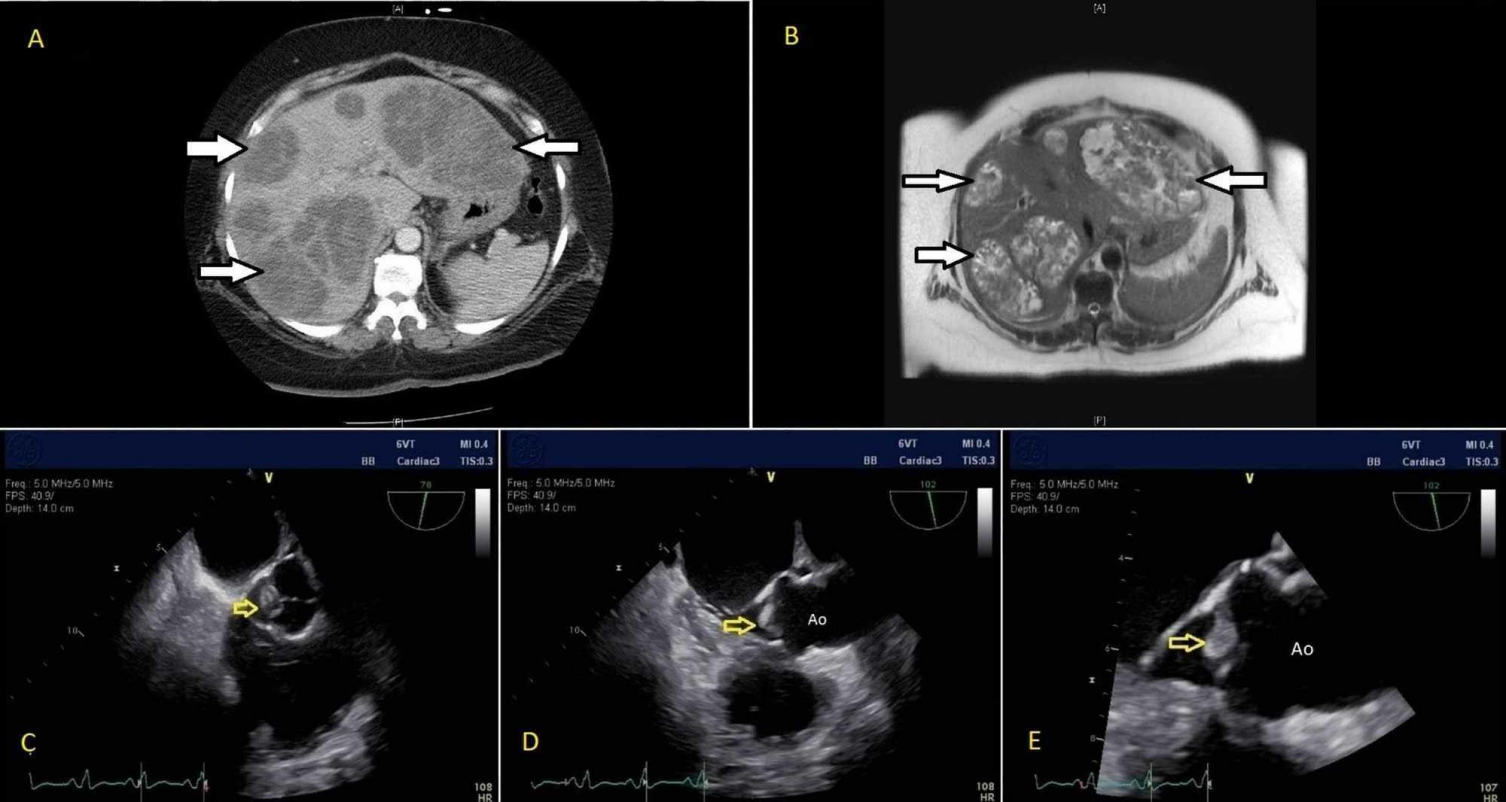
(A) CT abdomen and (B) MRCP finding of multiple hepatic abscesses (solid white arrows); (C-E) Transesophageal echocardiographic findings of aortic valvular vegetation (yellow arrows) Ao, Aorta; CT, Computed Tomography; MRCP, Magnetic Resonance Cholangiopancreatography

Initial lab results showed leukocytosis with left shift, normocytic anemia, elevated transaminases, hyperbilirubinemia, coagulopathy, and acute kidney injury (See Table 1 for lab results). She was immediately started on empiric broad-spectrum intravenous (IV) antibiotics after the initial blood cultures were drawn. Initial imaging included a liver ultrasound that revealed multiple hepatic lesions, confirmed with abdominal computed tomography (CT). Magnetic resonance cholangiopancreatography (MRCP) confirmed the absence of common bile duct dilation (See Figure 1A-1B for imaging findings). She had negative workup for both viral hepatides and tumor markers for gastrointestinal tumors. A CT-guided needle biopsy of hepatic lesions revealed purulent fluid with a white blood cell (WBC) count >170,000/mm^3^, negative for ova and parasites. The initial blood cultures drawn on admission and the fluid aspirate from hepatic lesions both grew Streptococcus intermedius (gram-positive cocci in clusters) with low penicillin minimal inhibitory concentration (MIC). The patient subsequently underwent a transesophageal echocardiogram revealing a one-centimeter aortic valvular vegetation without associated valvular dysfunction (Figure 1C-1E).

**Table 1:** Laboratory findings on initial presentation ALT, Alanine Transaminase; AST, Aspartate Transaminase; CRP, C - Reactive Protein; eGFR, effective Glomerular Filtration Rate; ESR, Erythropoietin Sedimentation Rate; INR, International Normalized Ratio; PT, Prothrombin Time; WBC, White Blood Cells

Laboratory findings			
WBC	15.82 K/mm^3^	Albumin	2.3 gm/dL
Creatinine	2.17 mg/dL	Total Protein	6.5 gm/dL
eGFR	29 ml/min	PT	16.5 sec
AST	387 unit/L	INR	1.57
ALT	235 unit/L	ESR	104 mm/hr
Total Bilirubin	3.3 mg/dL	CRP	28 mg/dL
Direct Bilirubin	2.2 mg/dL	Procalcitonin	21.59 ng/mL
Alkaline Phosphatase	108 unit/L		

The patient clinically improved and cleared blood cultures within 24 hours of abscess drainage and appropriate IV antibiotics. There was interval improvement in her renal and liver function tests, as noted in Table 2. She was discharged on intravenous antibiotics to complete a six-week course. Upon discharge, she was strongly recommended to take infective endocarditis prophylaxis during any future dental procedures involving mucosal manipulation and to take her antibiotic one hour prior to the procedure.

**Table 2:** Laboratory findings (improved) at the time of hospital discharge ALT, Alanine Transaminase; AST, Aspartate Transaminase

Interval improvement in pertinent laboratory findings		
Laboratory findings	On admission	On discharge
AST	387 unit/L	53 unit/L
ALT	235 unit/L	28 unit/L
Total Bilirubin	3.3 mg/dL	1.5 mg/dL
Direct Bilirubin	2.2 mg/dL	1.2 mg/dL
Creatinine	2.17 mg/dL	0.84 mg/dL

## Discussion

Streptococcus intermedius has been isolated from patients with periodontitis and can cause fatal purulent infections, especially brain and liver abscesses following dental procedures [7-9], but this is an unusual presentation culminating in endocarditis with an accompanying liver abscess. Usually, the most common Streptococcus (S) viridans group organisms known to cause IE are S. sanguinis, S. bovis, S. mutans, and S. mitisand the S. salivarius group that cumulatively accounts for the majority of native valve endocarditis [10-11] while S. intermedius (from the Streptococcus anginosus group) has a relatively lower incidence.

Although there have been limited cases of Streptococcus endocarditis documented after dental procedures, the most common clinical presentation after such procedures is that of bacteremia [12]. Transient bacteremia can occur after routine teeth brushing and does not necessarily require gingival manipulation. Reduction in bacteremia with antibiotic prophylaxis has not prevented infective endocarditis significantly [6]. Over the past decade (2006 - 2017), we have seen constant efforts by the American College of Cardiology (ACC) and American Heart Association (AHA) to address this clinical decision regarding IE prophylaxis. It has been shown that inappropriate antibiotic use in many cases can increase the risk of antibiotic resistance and there have been no randomized controlled trials performed to show the benefit of antibiotic prophylaxis in patients with a low risk of IE [2]. A nationwide population-based cohort and a case crossover study by Tubiana and colleagues suggest that invasive dental procedures may contribute to a very small number of cases of bacterial endocarditis, but the benefit of antibiotic prophylaxis remains uncertain and likely minuscule [13].

In this case, our patient did not meet the criteria for IE prophylaxis for the dental procedure. Her dental exam during this hospitalization revealed generalized mild periodontitis with no active abscess at the site of dental root extraction. However, despite receiving the IE prophylaxis prior to the dental procedure (amoxicillin two grams once), she developed endocarditis as the effective duration of action of administered antibiotic had already elapsed (half-life of 60 minutes with time to peak ranging from one to two hours and is excreted via urine within six to eight hours). Guidelines provide recommendations regarding the patient population at risk, the choice and the timing of such antibiotics (one hour before the procedure) [14]. Hence, it is a cornerstone step in explaining to patients the importance of prophylaxis timing (if indicated).

We take this opportunity to pose another critical question: how do we reduce the possibility of IE in low-risk patients without the use of antibiotics? A 2014 study by Elshibly et al. showed that simple oral hygiene with a total of one-minute exposure to high-potency mouthwashes killed viridans group streptococci organisms and suggested a significant reduction of IE [15]. A study by Veloso et al.used rats as the animal model for the induction of experimental endocarditis with continuous low-grade and intermittent high-grade bacteremia. It showed that both these mechanisms potentially induced experimental endocarditis. This data support the hypothesis that cumulative exposure to low-grade bacteremia (from longstanding inadequate dental hygiene) does represent a genuine risk of IE in humans [16]. However, these are single-center in-vitro studies lacking external validation and applicability to the large-scale human population. Regardless of individual risk factors, it is crucial to emphasize regular and thorough dental hygiene to all patients undergoing dental procedures. This is a low-level intervention without harm but may be beneficial, unlike antibiotic use for prophylaxis. Prophylaxis can only be an additive effect with daily dental hygiene for individuals with a high risk for IE.

 In the end, we pose several important questions through this case review:

1. What is the effective role of IE prophylaxis after all?

2. Mechanism of bacteremia, continuous low-grade (associated with inadequate regular dental hygiene) versus high-grade bacteremia (associated with dental procedures like gingival manipulation) or both?

## Conclusions

Transient bacteremia can commonly follow dental procedures but does not always necessarily require gingival manipulation. Reduction in bacteremia with antibiotic prophylaxis has not prevented IE significantly, but inappropriate antibiotic use (especially in low-risk patients) can potentially lead to antibiotic resistance. There have been no randomized controlled trials performed to show the benefit of antibiotic prophylaxis in low-risk patients, thus needing further studies in this perspective. A key concern is correctly identifying the population at risk and educating them about appropriate indications and the timing of effective antibiotic prophylaxis prior to the procedure. Until then, adequate dental hygiene on a regular basis is important to prevent continuous low-grade bacteremia (potential etiology for IE) to control viridans group streptococci. 
